# Considering internal conflict in the face of natural product biosynthesis and biosynthetic gene cluster evolution

**DOI:** 10.1042/EBC20250017

**Published:** 2026-08-19

**Authors:** John Bruce, Francisco Barona-Gómez, Paul A. Hoskisson

**Affiliations:** 1 Strathclyde Institute of Pharmacy and Biomedical Sciences, University of Strathclyde, 161 Cathedral Street, Glasgow, G4 0RE, U.K.; 2 Institute of Biology, Leiden University. Sylviusweg 72, 2333BE, Leiden, The Netherlands

**Keywords:** antibiotics, biosynthetic gene cluster, Streptomyces

## Abstract

The present essay attempts to stimulate interest and provide insight into the dynamics of internal conflicts, kin selection, and ecological interactions in multicellular, metabolically gifted microorganisms and how these processes may affect biosynthetic gene cluster (BGC) diversity. The multicellular antibiotic-producing soil bacterium *Streptomyces* provides a useful model for exploring how internal conflicts emerge and are resolved in biology. These organisms must balance two resource-intensive processes that can create internal conflicts—natural product biosynthesis and sporulation. In *Streptomyces*, there is potential to mitigate these internal conflicts through division of labour, phenotypic specialisation, and extensive gene duplication and diversification, enabling colonies to optimise both natural product production and reproductive success. Horizontal gene transfer further expands gene families and BGCs, introducing new metabolic capabilities while generating opportunities for functional divergence to reduce internal conflict and potentially promote kin selection. Natural product BGCs also possess features that could identify them as ‘greenbeards’ (kin selection by trait), promoting cooperation among producers and harming non-producers. The coexistence of multiple natural product BGCs and resistance mechanisms in *Streptomyces* is discussed in the context of the diverse eco-evolutionary processes occurring in structured natural environments, competition among close relatives, recurrent BGC acquisition, and regulatory compatibility encountered by *Streptomyces*.

‘*The forms which stand in closest competition with those undergoing modification and improvement will naturally suffer most.*’ Charles Darwin (1859), Origin of species.

## Introduction

The hierarchical organisation of complex life on earth arose from relatively few major evolutionary transitions—the emergence of operons, genomes, cells, multicellularity and interspecies symbiosis [1]. Within such systems natural selection is the primary mechanism that leads to adaptation [2,3], shaping genomes for the benefit of individual organisms. Yet, the existence of selfish elements in genomes undermines this view [4–6]. Selfish elements (segments of DNA that enhance their own transmission within a genome, such as transposons and insertion elements) create biological phenomena that can be interpreted according to the evolutionary interests of the selfish elements themselves, resulting in internal conflict [7]. Selfish elements can drive perpetual cycles of adaptation and counter-adaptation that shape genome evolution [4–6]. Internal conflict can, however, be considered in a broader context, with genetic elements, genes, and gene collectives giving rise to competing cellular processes. Remarkably, there is a lack of clarity and generalised definition of what internal conflict actually means [7] and how these internal conflicts apply and are resolved, with prokaryotic organisms receiving relatively little attention in this area. Here, internal conflict is considered as an evolutionary phenomenon where genes (or gene collectives) or cell types drive their own reproduction that may create conflict in overall organismal fitness [6].

It has been argued that for organisms to evolve adaptations, all of its parts must work towards the same end, with little within organism selection [8]. There is a resurgent interest in internal conflicts in biology, and how these may prevent organisms from maximising direct and indirect reproduction (inclusive fitness), such that adaptive phenotypes may exist at sub-organismal levels of selection [4,6]. In the present essay, we explore potential cellular conflicts from the perspective of the multicellular natural product (NP) producing bacterium *Streptomyces* and how these organisms may resolve such conflicts. We also explore whether there is a role for kin selection (the evolutionary strategy of boosting an organisms genetic success by increasing the fitness of genetic relatives), in the evolution of NP producing bacteria, ultimately driving the diversity of biosynthetic gene clusters (BGCs), pathways, and molecules we observe in *Streptomyces* strains.

## Produce and reproduce

The majority of clinically useful antibiotic compounds are produced by bacteria belonging to the genus *Streptomyces* and related genera within the *Actinomycetota* [9]. *Streptomyces* are characterised by their filamentous growth habit and their ability to form spores on specialised aerial hyphae produced in response to nutrient limitation and stress [10–12]. The vegetative hyphae of these organisms produce a plethora of bioactive NPs including antibiotics, used enhance competition with other organisms [13,14] along with other ‘public goods’ such as hydrolytic enzymes [12,13,15]. Whilst much of the focus on *Streptomyces* NPs has prioritised them based on their therapeutic applications as antimicrobials, anti-cancer, and immunosuppressives, it is likely that these molecules evolved as signals to mediate communication and interactions between organisms [9,16,17].

Entry into the developmental stage of the lifecycle in *Streptomyces* encompasses the simultaneous production of NPs, and unigenomic spore formation. Much is known about the sporulation process in *Streptomyces* [10,18,19] and about how NP production is regulated [20,21]. Both processes demand large amounts of the energy and biochemical building blocks arising from central metabolism: yet there are few studies of how cells reconcile the internal conflict arising from allocating resources to these two highly demanding processes. The aerial hyphae that give rise to spores are connected to the colony at a single point, possessing a hydrophobic cell layer [10], meaning that energy and cellular building blocks required to complete sporulation must be derived from the vegetative cells, which produce NPs, at a single contact point. These processes likely result in internal conflict in these multicellular organisms with NP production and sporulation competing for the same cellular resources, which may result in evolutionary trade-offs emerging [13].

The potential for internal conflicts in *Streptomyces* to arise through resource allocation competition between different cell types and NP production has resulted in several mechanisms known to resolve or modulate such conflicts being employed in *Streptomyces*. The specialisation and cooperation between cells in the form of division of labour [12,13,22], gene duplication, transcriptional specialisation, and diversification in primary metabolism are known to reduce potential conflicts [9,12,23–25].

Division of labour—where individuals specialise to carry out specific tasks and cooperation between phenotypes provides an inclusive fitness benefit to the group—may help resolve internal conflicts [26]. This has been considered in *Streptomyces*, where the clonal nature of colonies, derived from single unigenomic spores [26], may reduce the potential for the emergence of ‘cheater’ cells that may profit at the expense of the wider colony and undermine any division of labour [12,13,22,27]. In *Streptomyces*, the emergence within colonies of distinct and functionally specialised cells, with mutually exclusive phenotypes, is suggestive of a division of labour [13,26,28,29]. The multicellular nature of *Streptomyces* complicates the definition of what an ‘individual’ is given that each cellular compartment may contain 8–10 genetically distinct genomes [10,13]. Elegant work from the Rozen and co-workers [13] demonstrated that whilst early colonies are clonal in nature, the propensity for genetic instability in *Streptomyces* results in genetic heterogeneity emerging within colonies during maturation [13]. *Streptomyces* exhibit this genetic instability due to the frequent rearrangements, amplifications, and deletions that occur in these organisms [30–34]. Remarkably, these mutations can give rise to hyper-producing mutants, increasing levels of antibiotics throughout the colony, killing greater numbers of competitor cells, and increasing the fitness of the colony [22,35]. While the above example of division of labour appears to occur via terminal genomic differentiation, a second example of division of labour in *Streptomyces* is known, which is phenotypically plastic and spatially regulated [27], resulting in the production of different bioactive molecules in different locations in the colony. These two mechanisms are proposed to have evolved to reconcile the two main drivers of antibiotic (and other NPs) production in *Streptomyces—*that of competition sensing [36,37] and nutrient limitation [11].

Another remarkable feature of *Streptomyces* is that their genomes often possess multiple genes that encode the same putative biochemical function [24,25]. This expansion of gene families is achieved through gene duplication and the acquisition of additional copies of genes via horizontal gene transfer (HGT) [24]. The expansion of gene families in primary metabolism are thought to provide robustness to the genomes of *Streptomyces* and facilitate evolvability and adaptation [24,38,39], but in turn may facilitate internal conflict. The metabolic precursors for NP biosynthesis are derived from primary metabolism and many NP BGCs contain additional copies of primary metabolic genes [9,40]. This suggests that specific building blocks are processed by BGC specific metabolic enzymes to provision NP biosynthesis rather than primary metabolism. The presence of duplicate genes has long been debated by evolutionary biologists, with arguments suggesting they can create or ameliorate internal conflict [23]. Duplicate genes may result in internal conflict through antagonistic selection or reduce conflict through provision of additional gene copies that can diversify function. Gene duplication/expansion events can affect evolutionary conflict due to gene dosage effects, novel paralogs with conflicting expression patterns, and/or altered function and the mechanisms that have evolved to solve conflicts can vary widely [23].

One way to solve the evolutionary conflict of duplicate genes is through transcriptional specialisation (to a particular tissue type or lifecycle stage) [23]. In *Streptomyces*, the retention of expanded primary metabolic gene families does show specialisation in terms of transcription, with differential gene expression being common in primary metabolism [24,25], a phenomenon that can also be observed with spatial and temporal regulation of gene expression in duplicate copies required for development [41–43]. Solving intragenomic conflict of expanded gene families may also be achieved through neo- or sub-functionalisation of additional gene copies [44,45], although non-functionalisation remains the most likely route to solving intragenomic conflict [46]. Conflict generated through gene duplication events may lead to the emergence of antagonistic pleiotropy in cells, that is where fitness gains in one situation may negatively affect fitness under a different set of conditions, and functional diversification of duplicates may provide resolution to this conflict [23].

In bacteria, gene family expansion mostly occurs through HGT rather than gene duplication [47], and this could provide a mechanism to ameliorate conflict between additional gene copies through the arrival of sub-functionalised genes directly. The diversification of function can occur through altering enzyme kinetic parameters [46], reducing intragenomic conflict through changing the rate of the reaction (*V*), the specificity of the reaction (*K_m_*), the reaction turnover (*K_cat_*), the catalytic efficiency of the reaction (*K_cat_*/*K_m_*), and the nature of cofactor usage [23,46]. Yet, the partitioning of enzyme function through transcriptional changes, enzyme specificity and functional partitioning requires the action of selection to facilitate adaptation.

There is support for altered enzyme parameters in the literature, where detailed kinetic studies of expanded enzyme families have been undertaken in *Streptomyces*. A study of the duplicated glycolytic enzyme, pyruvate kinase (*pyk*) was found to have diversified enzyme *V, K_m_*, *K_cat_*, and cofactor usage [24]. Moreover, deletion of the differentially transcribed, duplicate genes also led to different effects on antibiotic production [24]. This was also observed in studies of duplicate phosphofructokinase enzymes in *Streptomyces* where duplicate genes were able to significantly affect central metabolic flux [48]. It is tempting to speculate that HGT provides the greatest source of gene expansion in bacteria because it reduces internal conflicts through reduced expression compared to gene-family members *in situ*, diminished protein-protein interactions and xenologs are not subject to the same regulatory conflict as paralogs, allowing more rapid evolution and diversification of function [47].

The presence of primary metabolic genes in NP BGCs is well established and believed to enhance the provision of substrates for biosynthesis [9]. The scenarios explored above appear to support the available literature, with duplication and HGT occurring in the expanded primary metabolic gene families in the scytonemin BGC in Cyanobacteria [49]. Clavulanic acid (CA) is biosynthesised from the condensation of arginine and glyceradehyde-3-phosphate. The product of the *argJ* gene in the CA producer *Streptomyces clavuligerus* has *N*-acetylornithinase activity, recycling the acetyl group from *N*-acetylornithine to L-glutamic acid, as the first step in arginine biosynthesis [50]. There are two additional copies of *argJ*, called *oat1* and *oat2*, located in the clavulanic CA BGC and in the paralogus CA cluster located on the large linear plasmid pSCL4 [50–52]. Enzymatic characterisation of ArgJ and Oat2 revealed diversification in *V* and *K_m_* suggesting that Oat2 supplements ArgJ activity when required during biosynthesis. Detailed enzyme kinetic studies are limited but the available data supports diversification of enzyme function and expression differences as a route to reducing internal conflict.

The potential reduction in internal conflict through division of labour, gene expression changes and gene expansion all seem to be employed by *Streptomyces* to cope with antagonistic cellular processes; however, a more thorough understanding is required and hopefully this discussion will help initiate experimental studies in the area.

## NP biosynthesis, resistance, and ‘greenbeards’

BGCs in *Streptomyces* generally adhere to a common framework regarding their architecture—they are usually contiguously organised on the chromosome and possess structural genes for biosynthesis, transport genes for the export of the product and genes responsible for regulation [9,40,53]. Additionally for antibiotics (and other toxins) there are often immunity/resistance genes, to ensure that self-killing does not occur, often an export function, or through target duplication elsewhere in the chromosome [53]. Studies of bacteriocin producing bacteria show that producing antibiotics and constitutive resistance are costly to the producers, and the benefits of antibiotic biosynthesis may also accrue to others [54].

**Figure 1:**
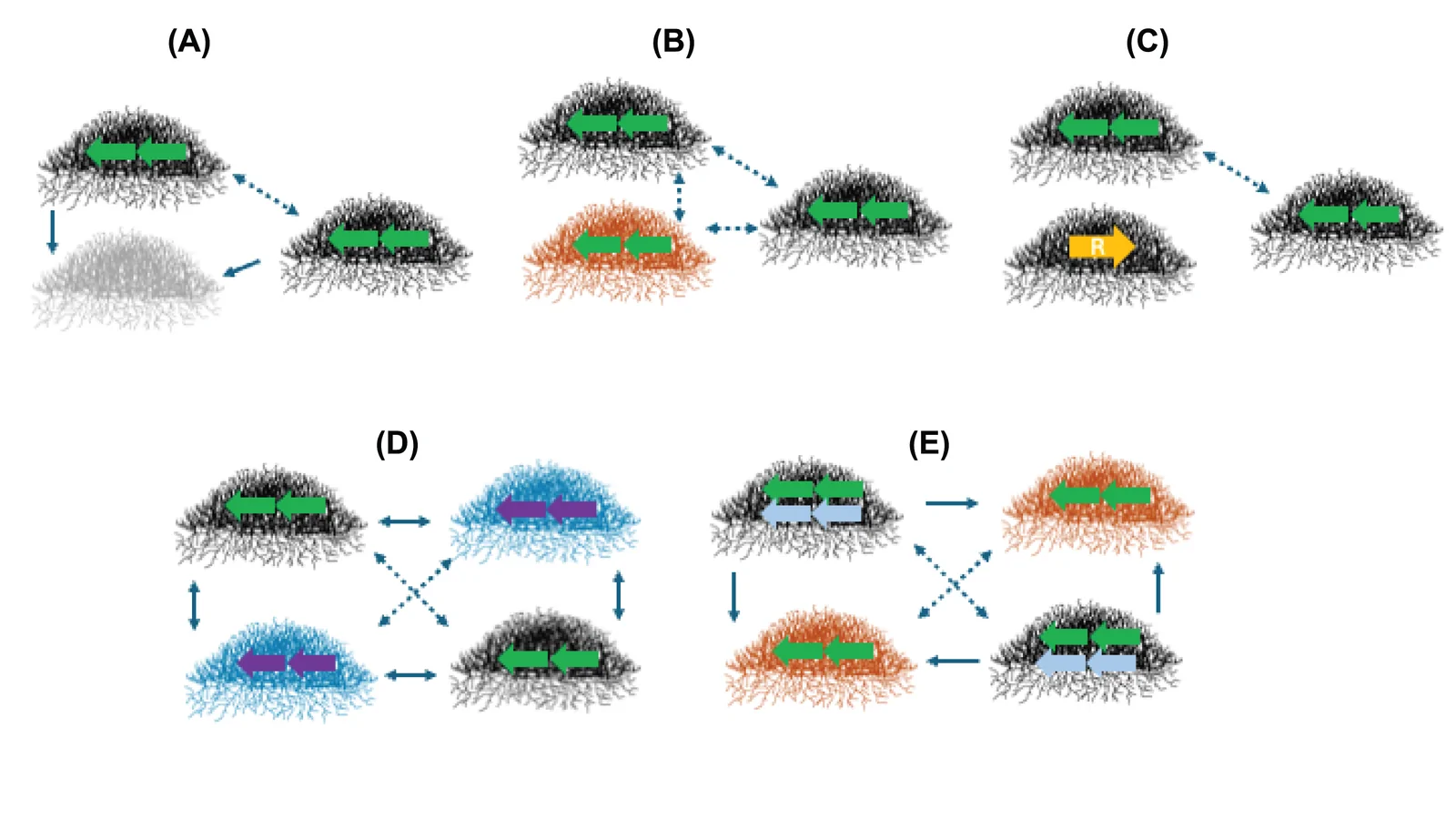
NP BGCs in *Streptomyces* as greenbeards BGC clusters depicted as coloured arrows, the independent resistance gene is a single yellow arrow. Dotted double-ended arrows indicate reciprocal production and resistance. Solid double-ended arrows indicate potential for reciprocal harm. Single ended solid arrows indicate the direction of harm. (**A**) Non-beards—*Streptomyces* colonies (black) containing an antibiotic BGC (green arrows), signalling to other greenbeard *Streptomyces* colonies, when in competition with a ‘non-beard’ strain (grey) that does not share the same BGC, which can be killed/outcompeted. (**B**) Greenbeards—*Streptomyces* colonies (black) containing an NP BGC (green arrows), signalling to other greenbeard *Streptomyces* colonies (orange), which are not genealogical kin, but share the same greenbeard trait—the NP BGC. (**C**) Falsebeards—*Streptomyces* colonies (black) containing an antibiotic BGC, signalling to other greenbeard *Streptomyces* colonies, when in competition with a ‘falsebeard’ strain that does not share the same BGC, but possesses a resistance gene (yellow ‘R’ arrow). The falsebeard receives the benefit of the greenbeard strain producing an NP, but bares none of the costs of production. (**D**) Multicoloured greenbeards—*Streptomyces* colonies (black) containing an NP BGC, signalling to other black greenbeard *Streptomyces* colonies, when in competition with strains that exhibit a different coloured ‘greenbeard’ trait (blue colonies) that does not share the same BGC, but produces a different antibiotic (purple arrows), creating competition. (**E**) Greenbeard-plus—*Streptomyces* colonies (black) containing two NP BGCs (green arrows and pale blue arrows), signalling to other greenbeard *Streptomyces* colonies. While the orange colonies share production and resistance to one NP (green arrows), they lack the additional (pale blue) NP BGC. The black colonies that have acquired an additional BGC allows them to drive their own inclusive fitness interests even when a BGC is shared. It is hypothesised that this mechanism may drive BGC acquisition in *Streptomyces*. *Streptomyces* colony image courtesy of Phylopic.org CC0 1.0.

The concept of the ‘greenbeard’ was developed to explain a specific kind of kin selection [6,55]. Greenbeards are genes or clusters of linked genes that can identify copies of themselves in other individuals and cause the barer to act nepotistically towards those individuals [8,56–58], directing help towards other carriers (greenbeards) and harm to non-carriers (non-beards; Figure 1A) [54]. The greenbeard effect is different to other mechanisms of kin selection, as it does not necessarily identify genealogical kin but simply other carriers of the greenbeard trait [57] (Figure 1B). Greenbeards are difficult traits to identify as they approach fixation within a population [58]. This can happen rapidly in prokaryotes, where extensive HGT, high reproductive rates and large population sizes result in rapid spread of beneficial genes in populations [59,60], such that discrimination between greenbeards maybe lost. However, several examples have been identified in bacteria where cheating strains (or ‘falsebeards’; Figure 1C) emerge that can receive the benefit of the greenbeard behaviour, yet bare none of the cost of performing the behaviour [8,54,57,58]. In *Streptomyces*, these strains may acquire resistance to an NP and coexist with producing strains. It is proposed NP BGCs may be considered as greenbeards, with some of the best characterised NPs acting as toxins yet there are signalling and competitive roles proposed for other NPs such as siderophores, butyrolactones etc [17] where production, secretion and uptake can be costly. Given that the natural role of many NPs is poorly understood [9,17] much of the following discussion is focussed on antibiotic production, but the same arguments could hold for a range of NPs.

When considered in the context of antibiotic production in *Streptomyces* there are several features of biosynthesis and resistance characteristic of greenbeards: they invest heavily in production, both in terms of genome content and resource provision, as biosynthesis and secretion are costly to the producer, but the benefits (death of competitors) are shared with others that perform the behaviour (and potentially some who do not—see below). Resistance in producers is also costly, with strains investing in resistance in a number of ways such as within cluster resistance, cluster-independent resistance and target duplication [53]. In *Streptomyces*, the existence of cluster-independent resistance allows non-producing strains to benefit from producing strains without the cost of producing themselves—so called ‘falsebeards’. The emergence of cheater strains has not been studied in this context in *Streptomyces*, yet it is known that many strains occurring in nature possess resistance to antibiotics they do not produce, such as chloramphenicol resistance in *Streptomyces coelicolor* and, extensive beta-lactam and aminoglycoside resistance in many strains [13,61]. In a recent study, cheater-like strains have been shown to emerge in communities of *Streptomyces* species associated with siderophore production [62], suggesting that interspecific cheating (siderophore piracy) can occur in *Streptomyces*. This aligns well with the bacteriocin model of Biernaskie et al. [54], but as discussed by these authors, what happens when multiple beard colour-types (antibiotics) exist, how is diversity maintained, what dynamics drive this, and whether this promotes diversity in modes of action of NPs remain unclear.

To understand how multicoloured greenbeards arise and are maintained, the bacteriocin modelling work of Biernaskie et al. [54] showed that resistant strains (falsebeards) can promote the maintenance of multiple bacteriocins and immunity types in populations, even when competing strains differ in their toxicity. This appears to mimic what we know about NP production in *Streptomyces—*where multiple BGCs can exist in a strain encoding similar biological activities (targeting the same cellular processes) and immunity may be achieved in several ways [9]. Coexistance of greenbeard and falsebeard strains is further facilitated in Biernaskie et al. [54] in structured environments (such as soil, where the majority of *Streptomyces* have been isolated) suggesting potential for this model to be experimentally tested.

In *Streptomyces* it is also well established that strains are capable of producing a range of antibiotics (multicoloured greenbeards; Figure 1D) and possessing multiple resistance mechanisms [63–68]. The modelling of Biernaskie et al. [54] also showed that selection likely acts when diversity is high, where multiple bacteriocin-types or immunity mechanisms are present in a community of organisms [54]. How bacteriocin diversity is generated and maintained is not well understood in nature. One way to achieve this, however, is via HGT and it is well established that antibiotic BGCs are frequently transferred between strains by HGT [69,70], with several BGCs contained on large plasmids in *Streptomyces* (e.g [52,71,72]).

Studies of *Streptomyces* community dynamics in the natural environment are rare and difficult to undertake in truly natural conditions [73], however, it has been shown that the non-random community structure of *Streptomyces* in soil communities can be explained by the inhibition and resistance characteristics of the community members [74]. Indeed, it is well known that phylogenetically distinct *Streptomyces* are capable of producing the same NPs [75,76], and given BGCs are tightly regulated and expressed in a highly context dependent manner [11,20,77,78] it is likely that not all NPs are made in each ecological niche. Intriguingly, studies such as those from Vetsigian et al. [67,79] indicate that stable communities of *Streptomyces* can be formed with different NP biosynthetic capabilities and antagonism is possible when conditions change. This suggests that diversity of NP production (particularly antibiotics) and resistance to antibiotics is maintained and expanded through competition, but ecological context matters [74].

There does however appear to be mechanisms that constrain the carrying capacity of NP BGCs in these organisms. There is not a simple continuous co-evolutionary arms race between organisms with continuous BGC acquisition [71,72], expanding the NP repertoire and genome size, suggesting that there would be a dynamic acquisition and loss of BGCs in *Streptomyces* genomes. The functional replacement of BGCs in *Salinospora*, a relative of *Streptomyces*, suggests that there is locus specific conflict, where replacement of BGCs with another BGC that has a similar mode of action, provides a potential mechanism to reduce redundancy and fitness costs associated with increased genome size and replication [80]. Moreover, ecological competition and selection drive diversification in *Salinospora* [81], suggesting that acquisition and replacement of BGCs could be a frequent mechanism to maintain BGC diversity and drive clonality in antibiotic producers. However, this does not explain the capacity for production of multiple antibiotics, with similar activities, and other NPs in *Streptomyces* and their relatives. Perhaps a driver for this is that antibiotics are beneficial to producing organisms because they bind cellular targets in competitor organisms, although relatively few cellular processes are targeted by antibiotics [53]. The ‘target-based’ model of antibiotic evolution described by Fischbach and Clardy [82] along with functional replacement suggests a role for maintenance of target diversity rather than chemical or pathway diversity.

In *Streptomyces*, the presence of multiple antibiotic BGCs with related activities occurs frequently, and within-species antibiotic diversity is common [9,83]. This moves beyond the ‘multicoloured greenbeards’ scenario where a single species possesses one (or few) variants of many kinds of NP, to a single species possessing several kinds of NP driven by biochemical target diversity. This hypothesis remains to be tested, but the wealth of genomic data and tools available make this an attractive problem to approach and it is hoped that the present essay will help stimulate interest in this area.

It is not known what drives the acquisition and maintenance of more than one BGC with similar activities in *Streptomyces*, but several aspects of their biology may provide clues. It has been recognised since Darwin [84] that competition between closely related species is likely to be the most intense, and given the abundance of *Streptomyces* in soil, it would be expected that competition with other *Streptomyces* species for resources is frequent and intense. The limited experimental data available also supports this view, with antibiotic inhibition being more intense between sympatric than allopatric *Streptomyces* populations [73,74]. Gene exchange within closely related *Streptomyces* species is frequent and provides the raw material for recombination and innovation in BGC evolution [53]. Moreover, HGT of complete BGCs in closely related species allows for exploitation of existing regulatory networks in new hosts, potentially reducing internal conflict over host resources. Other mechanisms such as BGC co-operation/cross-regulation (examples in [9]) provide further opportunity to reduce internal conflict and facilitate BGC maintenance. Mechanisms such as feed-forward regulation and antibiotic activated resistance/export also may facilitate BGC fixation in populations by triggering organisms to produce the antibiotic and activate resistance [85–89].

The HGT of complete and functional NP BGCs may also create scenarios where successive BGC acquisition by strains can help organisms to discriminate more easily between kin and non-kin cells, potentially harming those that lack the same complement of BGCs. This scenario (Figure 1E) could lead to repeated acquisition or replacement of bioactive NP BGCs within strains, where the acquisition of new BGCs or functional replacement of BGCs allows cells to compete with those that are not genetically identical. Experimentally testing this hypothesis in an ecologically relevant, structured environment is technically challenging, but innovative studies such as Tidjani et al. [90] and Vetsigian et al. [67] can help give insight into how such studies could be undertaken. There is still much to learn about the mechanisms that drive BGC maintenance and diversity in these organisms and it is hoped the present essay will stimulate attempts to experimentally study some of these hypotheses.

## Summary

- Antibiotic-producing organisms have to reconcile two-resource intensive processes that can create internal conflicts—antibiotic production and sporulation.
- *Streptomyces* may mitigate internal conflicts through division of labour, phenotypic specialisation, and extensive gene duplication and diversification that enable colonies to optimise both antibiotic secretion and reproductive success.
- Antibiotic BGCs also have features that identify them as ‘greenbeards’, promoting cooperation among producers and harming non-producers.
- Coexistence of multiple antibiotics and resistance mechanisms in *Streptomyces* likely reflects eco-evolutionary processes involving structured environments, competition among close relatives, recurrent BGC acquisition, and regulatory compatibility.

## Open Access

Open access for this article was enabled by the participation of the University of Strathclyde in an all-inclusive Read & Publish agreement with Portland Press and the Biochemical Society under an agreement with JISC.

## Abbreviations

BGC: biosynthetic gene cluster
CA: clavulanic acid
HGT: horizontal gene transfer
NP: natural product
